# South‒South research collaborations in critical care

**DOI:** 10.62675/2965-2774.20250156-2

**Published:** 2025-07-31

**Authors:** Alexandre Biasi Cavalcanti, Moses Siaw-Frimpong, Daniela Carla de Souza, Glenn Hernandez, Madiha Hashmi, Jorge Ibrain Figueira Salluh

**Affiliations:** 1 HCor-Hospital do Coração Research Institute São Paulo SP Brazil Research Institute, HCor-Hospital do Coração - São Paulo (SP), Brazil.; 2 Brazilian Research in Intensive Care Network São Paulo SP Brazil Brazilian Research in Intensive Care Network (BRICNet) - São Paulo (SP), Brazil.; 3 Latin-American Intensive Care Network Santiago Chile Latin-American Intensive Care Network (LIVEN) - Santiago, Chile.; 4 Komfo Anokye Teaching Hospital Department of Anaesthesia and Intensive Care Kumasi Ghana Department of Anaesthesia and Intensive Care, Komfo Anokye Teaching Hospital - Kumasi, Ghana.; 5 Kumasi Centre for Collaborative Research into Tropical Medicine Global Health and Infectious Disease Group Kumasi Ghana Global Health and Infectious Disease Group, Kumasi Centre for Collaborative Research into Tropical Medicine - Kumasi, Ghana.; 6 Universidade de São Paulo Hospital Universitário Department of Pediatrics São Paulo SP Brazil Pediatric Intensive Care Unit, Department of Pediatrics, Hospital Universitário, Universidade de São Paulo - São Paulo (SP), Brazil.; 7 Pontificia Universidad Católica de Chile Facultad de Medicina Departamento de Medicina Intensiva Santiago Chile Departamento de Medicina Intensiva, Facultad de Medicina, Pontificia Universidad Católica de Chile - Santiago, Chile.; 8 Ziauddin University Department of Critical Care Medicine Karachi Pakistan Department of Critical Care Medicine, Ziauddin University - Karachi, Pakistan.; 9 Instituto D’Or de Pesquisa e Ensino Rio de Janeiro RJ Brazil Instituto D’Or de Pesquisa e Ensino - Rio de Janeiro (RJ), Brazil.

Approximately 85% of the world's population lives in low- and middle-income countries (LMICs), i.e., the Global South. The burden of critical illness is disproportionately greater in the Global South, with a greater incidence of intensive care syndromes and poorer in-hospital outcomes than in high-income countries (HICs).^(1,2)^ Despite this substantial burden, only 22% of critical care publications originate from LMICs.^(3)^ Large international studies involving intensive care patients are predominantly led by researchers based in HICs, whereas studies led by LMIC-based researchers—especially those involving collaboration between HICs and LMICs—are rare.^(4)^

## HIGH-INCOME COUNTRIES-LED INTENSIVE CARE RESEARCH

**Figure 1:**
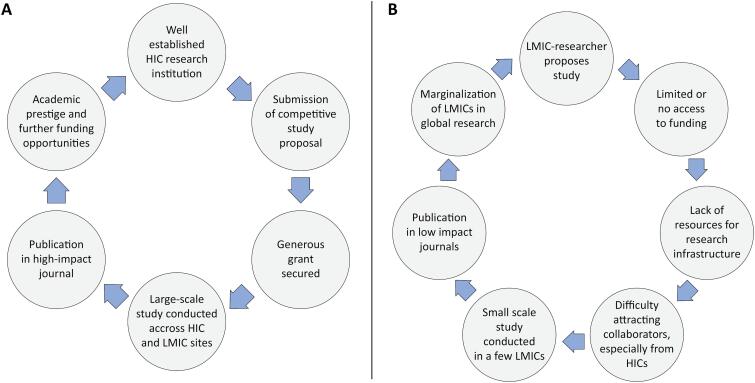
Cycle perpetuating high income country-based leadership of large international intensive care studies (A) and cycle limiting prospects of low-and-middle income countries in global research leadership (B).

The current model of international collaborative research tends to favor leadership by researchers based in HICs, a pattern that may be driven by several factors.^(5)^ First, research funding is more abundant in HICs, where most agencies that support international studies are based. These agencies may preferentially fund researchers from their own regions. Second, a virtuous cycle exists in which the successful funding, execution, and publication of international studies further strengthens academic research organizations in HICs and reinforces the track records of HIC-based investigators (Figure 1A). In contrast, the lack of funding and support for researchers in LMICs severely limits the development of research infrastructure and leadership capacity, perpetuating existing disparities (Figure 1B). This scenario also results in a "brain drain" from LMICs, resulting in the transfer of LMIC-based researchers to HICs. Third, when LMIC-based researchers attempt to initiate collaborations and invite HIC-based leaders to participate in international research efforts, the response is often negative (as reported by the coauthors’ personal experience), reflecting a lack of reciprocity. This reluctance may be attributed to limited funds to support HIC sites, less established academic profiles of LMIC investigators, and perhaps a subjacent culture of academic dominance in research. Fourth, authorship practices tend to favor HIC researchers. Scientific publishing remains largely centered in the Global North, with limited representation of LMICs.^(6)^ The top 50 medical journals are, without exception, based in North American and European countries.^(6)^ In collaborative international studies, LMIC researchers are often relegated to middle author positions, whereas first and senior authorship is disproportionately held by those from HICs.^(7)^

HIC - high income country; LMIC - low-and-middle income countries.

## SOUTH-SOUTH INTENSIVE CARE RESEARCH COLLABORATION

Expanding the capacity to conduct intensive care research in LMICs is essential. To achieve this widely agreed-upon goal, the infrastructure and expertise needed to coordinate large-scale studies within LMICs need to be expanded. To address this, many efforts have been made through North‒South collaboration to increase the capacity of research in LMICs. However, these North–South partnerships face significant challenges such as poor role clarity, power imbalance, and lack of due recognition.^(8)^ We propose that one promising strategy to overcome the barriers faced by LMICs is to foster collaborative research initiatives among Global South research networks.

Why do intensivists from the Global South need to engage in South–South research collaborations? One compelling reason is the potential for greater reciprocity. These collaborations are more likely to be equitable, as researchers from participating countries can realistically expect mutual support when initiating new studies. Moreover, South–South collaboration can facilitate the conduct of large, high-impact studies addressing questions that are relevant to LMIC settings. By generating locally applicable evidence, these studies can help prevent the unnecessary adoption of costly therapies supported by low-quality evidence, resulting in meaningful cost savings for already resource-constrained health systems. This scientific output can, in turn, enhance the competitiveness of LMIC research groups in securing funding from major international agencies.

## WHERE WE ARE: CHALLENGES AND EXAMPLES

Several barriers still limit the full development of collaborative research among Global South countries.^(9,10)^ These include limited funding, a precarious research infrastructure, and fewer opportunities for research training. Additionally, the lack of well-equipped laboratories, restricted access to databases, and technological disparities between countries hinder the execution of high-quality multicenter studies. Navigating diverse ethical review processes and regulatory frameworks across multiple LMIC contexts creates significant administrative burdens and study delays. Language and cultural barriers hinder the scientific output of the Global South and its indexing in high-impact journals, which limits the visibility of findings and their adoption in global guidelines.^(4,11)^

Due to the limited availability of recognized medical journals based in LMICs, researchers often have to submit their work to journals in high-income countries, where implicit biases may sometimes pose additional barriers to publication. This gap can leave researchers vulnerable to predatory journals that charge high fees without providing rigorous peer review. In this context, journals such as Critical Care Science, which is based in a Global South country and offers free access for both authors and readers, represent a highly valuable platform for disseminating high-quality research from LMICs. Increasing its visibility and recognition within critical care societies would further strengthen its role in promoting equitable global research dissemination. Skilled human resources are essential for establishing a robust research system capable of sustaining South–South collaboration. Achieving this goal requires a coordinated effort to identify and complement the strengths and gaps in research capacity across participating countries. Ongoing training and mentorship initiatives are also crucial to ensure the development of a sustainable research workforce for future generations.

Another challenge in the development of South–South collaborations is ensuring that all participant countries benefit equally, limiting skewed growth of certain LMICs at the expense of others. It is essential to prevent the replication of an academic dominance research model within LMIC partnerships. Fair and clear guidelines are needed to foster a sustainable partnership, with decentralized leadership and decision-making processes.

The International Severe Acute Respiratory and Emerging Infection Consortium (ISARIC), a global research network established to prepare for and respond to outbreaks of infectious diseases, is a valuable example for developing research in LMICs.^(12)^ Although including both North and Global South countries, the consortium has promoted global equity in research, with a particular focus on enabling LMICs to contribute data and lead studies. ISARIC supports capacity-building, training, and flexible core funding models, which foster locally led investigations during health emergencies.

Successful South‒South research networks include, the Latin American Intensive Care Network (LIVEN), the African Critical Care Collaboration (ACCC), the Asian Critical Care Clinical Trials Group, and the Collaboration for Research, Training, and Implementation in Critical Care in Asia and Africa (CCAA).^(13-15)^ Brazilian Research in Intensive Care Network (BRICNet), conversely, includes only Brazilian ICUs, although it has collaborated with other networks, such as the LIVEN or Australian New Zealand Intensive Care Society (ANZICS).^(16)^ All these Global South networks have produced significant studies and supported guidelines for the treatment of critically ill patients. For example, the CCAA network, which managed to obtain substantial international funding, transformed critical care research in over 18 LMICs, significantly enhancing clinical decision-making and research capacity across intensive care units in these regions.^(13)^ Fully global South-to-South intensive care research collaboration is the next step forward.

## MAKING IT HAPPEN: NEXT STEPS FOR EFFECTIVE COLLABORATION

Certainly, feasibility is a key concern—particularly because initial Global South–led collaborations are likely to have limited or no funding. Fortuitously, LMIC sites are often more willing to participate in LMIC-led observational studies and randomized trials, even when budgets are minimal. This willingness creates a unique opportunity: large academic studies led by and involving LMIC intensive care networks are not only feasible but also have the potential to disrupt the current model of HIC-led international research.

## CONCLUSION

Collaboration among Global South countries to conduct large-scale intensive care studies provides a powerful solution, with the potential to disrupt the prevailing model of HIC-led research, strengthen critical care research capacity and improve patient outcomes in LMICs.
